# Insights into the Influence of Priors in Posterior Mapping of Discrete Morphological Characters: A Case Study in Annonaceae

**DOI:** 10.1371/journal.pone.0010473

**Published:** 2010-05-10

**Authors:** Thomas L. P. Couvreur, Gerrit Gort, James E. Richardson, Marc S. M. Sosef, Lars W. Chatrou

**Affiliations:** 1 The New York Botanical Garden, New York City, New York, United States of America; 2 Biosystematics Group, Netherlands Centre for Biodiversity Naturalis (Section Nationaal Herbarium Nerderland), Wageningen University, Wageningen, The Netherlands; 3 Biometris, Wageningen University, Wageningen, The Netherlands; 4 Royal Botanic Garden Edinburgh, Edinburgh, United Kingdom; 5 Biosystematics Group, Wageningen University, Wageningen, The Netherlands; University College London, United Kingdom

## Abstract

**Background:**

Posterior mapping is an increasingly popular hierarchical Bayesian based method used to infer character histories and reconstruct ancestral states at nodes of molecular phylogenies, notably of morphological characters. As for all Bayesian analyses specification of prior values is an integrative and important part of the analysis. He we provide an example of how alternative prior choices can seriously influence results and mislead interpretations.

**Methods/Principal Findings:**

For two contrasting discrete morphological characters, namely a slow and a fast evolving character found in the plant family Annonaceae, we specified a total of eight different prior distributions per character. We investigated how these prior settings affected important summary statistics. Our analyses showed that the different prior distributions had marked effects on the results in terms of average number of character state changes. These differences arise because priors play a crucial role in determining which areas of parameter space the values of the simulation will be drawn from, independent of the data at hand. However, priors seemed to fit the data better if they would result in a more even sampling of parameter space (normal posterior distribution), in which case alternative standard deviation values had little effect on the results. The most probable character history for each character was affected differently by the prior. For the slower evolving character, the same character history always had the highest posterior probability independent of the priors used. In contrast, the faster evolving character showed different most probable character histories depending on the prior. These differences could be related to the level of homoplasy exhibited by each character.

**Conclusions:**

Although our analyses were restricted to two morphological characters within a single family, our results underline the importance of carefully choosing prior values for posterior mapping. Prior specification will be of crucial importance when interpreting the results in a meaningful way. It is hard to suggest a statistically sound method for prior specification without more detailed studies. Meanwhile, we propose that the data could be used to estimate the prior value of the gamma distribution placed on the transformation rate in posterior mapping.

## Introduction

Bayesian inference of character evolution is a novel way to map characters along phylogenies [1], [2], [3], [4]. It attempts to summarize unobserved character histories that could have given rise to the observed data on the tips of a phylogeny. A character history reveals more information about the evolution of a specific character than just the reconstruction of ancestral states at the nodes of the tree. Additionally, it provides information about the number of changes, the timing and placement, and the type of change that occurred along the tree(s) [1], [4]. In contrast to the widely used maximum parsimony optimization method, which optimizes characters by minimizing the number of state changes across a fixed topology (or on a sample of topologies, e.g. MrBayes), the Bayesian approach simultaneously accommodates for both mapping as well as phylogenetic uncertainty, i.e. alternative reconstructions within and between equally likely trees respectively [4], [5], [6]. In addition, it also allows for character states to change along a branch instead of just at the nodes, which is especially important for long branches for which the probability of change is much higher [1], [4], [7]. Two main methods of Bayesian inference of character evolution have been advanced differing generally by the underlying model of trait evolution: that of Pagel et al. [2] and that of Huelsenbeck et al. [4]. In this study we shall focus on the latter method termed posterior mapping as introduced by Huelsenbeck et al. [4]. Posterior mapping was originally developed for DNA sequence data [3] but its use has since been extended to morphological characters [4]. For discrete morphological characters, which are the main focus of this study, the posterior mapping approach using a continuous-time Markov chain and implementing the Mk model of Lewis [8] has been proposed [1], [4]. The continuous-time Markov chain contains a transition matrix defined by two parameters: the rate of transformation of the morphological character (θ) and a bias parameter governing the direction of change between each character state (Л). These Markov evolutionary model parameters (θ and Л) are drawn from their posterior distribution. The prior probability distribution of the rate of transformation θ is modelled as a gamma distribution with parameters α_S_ and β_S_, while a beta distribution with parameters α_B_ and β_B_ is placed on the directional bias Л. In both cases the values of the parameters α and β refer to the shape and inverse scale parameters of the gamma distribution defining the mean (E) and the standard deviation (SD) of the distributions [4], [9]. The characterisation of two prior values (directional bias Л and rate of transformation θ) is the main difference with the other method of Bayesian inference of character evolution as introduced by Pagel et al. [2]. Indeed, in the Pagel et al. method there is only one rate parameter that is modelled as a beta distribution [2]. Finally, ancestral state characters are then estimated based on their marginal posterior probability [4], which is calculated by integrating over the uncertainty in all of the other model parameters (tree topology, branch lengths, etc.).

As for all Bayesian analyses specifying prior values can be problematic and many researchers feel uneasy in doing so [10], [11], [12]. This apprehension could come from a lack of understanding of the effect of the priors on the final results. Moreover, in recent studies that apply posterior mapping to study the evolution of morphological and ecological characters, the values of the parameters are generally not reported [13], [14], [15], [16], [17] or their impact on the final results is not addressed [18]. The nature of the beta distribution placed on the directional bias prior (Л) allows for the use of a so-called flat or uninformative prior (α_B_ = β_B_ = 1). Probabilities are uniform over the whole parameter space providing an adequate and widely used alternative to the lack of prior knowledge. For this study the influence of the prior on the directional bias (Л) will not be addressed. In contrast, and most importantly, the gamma distribution placed on the rate of transformation θ cannot accommodate for uniform priors. Any combination of the two parameters, α_S_ or β_S_, will define the mean E(T) and the standard deviation SD(T) of the prior distribution. For morphological character evolution, the impact of this prior distribution on the realizations has received meagre attention and to our knowledge has not been thoroughly assessed using empirical data. Schultz and Churchill (1999), using simulated data, showed that certain combinations of priors on θ and Л can influence the outcome of simulations. Huelsenbeck et al. [4], applied different transformation rate priors, a slow and fast mean rate E(T), with a flat prior on the directional bias (Л), on two different empirical datasets. They noticed that the posterior probabilities of the character histories were independent of the prior used. How the priors affect the outcome of the realizations in terms of average number of transformations and the posterior probability of a character history remains unclear. With the advent of user-friendly software like SIMMAP [19], enabling a more widespread application of this method, it is important to renew awareness of this issue.

**Figure 1:**
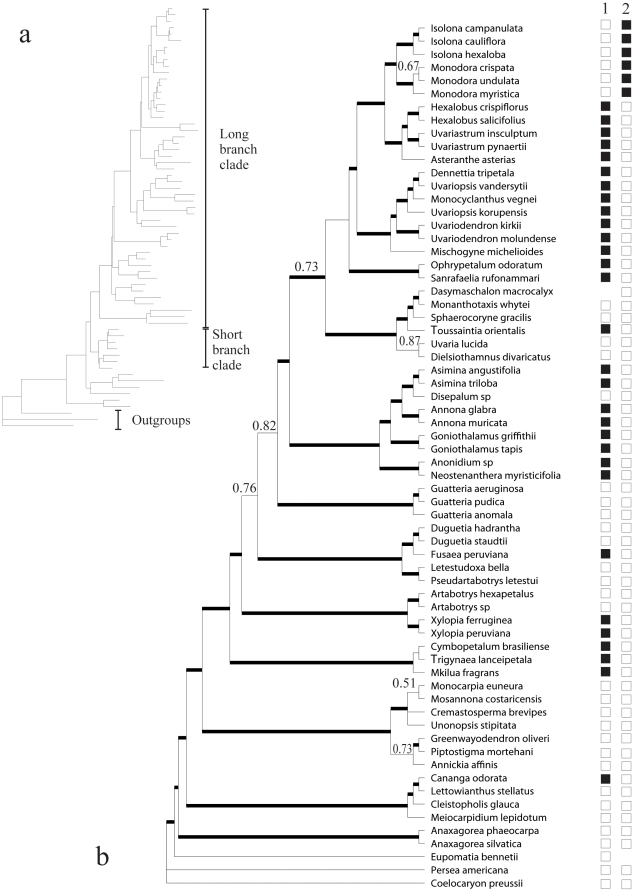
Phylogenetic relationships within the long branch clade in Annonaceae. a) Phylogram of the majority rule consensus tree of the last 30,000 trees sampled after five million generations of the MCMCMC run indicating the branch lengths as well as the two major groups recognized within the Annonaceae: long branch and short branch clade. b) Majority rule consensus tree of the last 30,000 trees sampled after five million generations of the MCMCMC run. Posterior probabilities under 0.95 are displayed at nodes. Thick branches indicate support >0.95 PP. The distribution of species with compound pollen (1, black squares) and syncarpy (2, black squares) are represented along the tips of the phylogeny. Missing squares indicate absent observations; the species was scored as uncertain for that character.

To this end we undertook an empirical study of two morphological characters found within the flowering plant family Annonaceae of the early diverging magnoliids [20]. Recent molecular phylogenetic studies [21], [22], [23] revealed a well supported clade with on average twice the level of sequence divergence (the so-called long branch clade, LBC) when compared to a second major clade with lower levels of sequence divergence (the so-called short-branch clade). The LBC is generally characterized by long branches (Figure 1 a) subtending species-rich clades [24]. The long branches of the LBC offer an ideal situation for applying posterior mapping to the study of the evolution of morphological characters, given the flaws that might be expected when applying maximum parsimony optimization to character reconstruction. Two contrasting morphological characters found within the LBC were selected (Figure 1 b). (1) A potentially slow evolving character, carpel fusion, which has two states: apocarpy and syncarpy. Syncarpy is defined as the congenital fusion of the female reproductive units of the flower termed carpels [25], [26], and has only rarely evolved within the magnoliids. In Annonaceae, however, syncarpy has evolved in the ancestor of two strongly supported African sister genera *Isolona* and *Monodora* [25], [27], [28]. Syncarpy was specifically chosen because one can ‘intuitively’ infer from the tree that this character evolved once, and thus allows us to evaluate in a more informed way how different priors can or can not influence the results. (2) A potentially faster evolving character: pollen unit, again with two states: single (pollen composed of a single grain) and compound (pollen composed of two, four or numerous grains). The single state is considered ancestral within Annonaceae with reversals being fairly common [29].

The aim of the present study was to investigate, within Annonaceae and for the two characters described above, how prior selection of the transformation rate θ can influence certain important values (e.g. the average number of transformations or the marginal posterior probability of each ancestral character states) by subjecting empirical data to the posterior mapping method. Thus, this study was not designed to compare results between alternative methods of character optimization. For such comparisons the reader is referred to Huelsenbeck et al. [4] or Ekman et al. [30].

## Results

### Phylogeny

The partition strategy strongly supported under the Bayes factor was run for five million generations with three independent runs. The posterior probabilities of all splits were indistinguishable between independent runs as visualized with AWTY (results not shown), suggesting convergence between them. In addition, all three runs reached stationarity after 250,000 generations with all of the parameters converging to the same values as visualized with Tracer. The majority rule consensus tree was generally well resolved and well supported (Fig. 1). For details about the MrBayes analysis and discussion about the phylogenetic relationships in Annonaceae resulting from this analysis see Couvreur et al. [28].

### Influence of the Rate Prior θ

The average number of total transformations as well as the average number of transformations from one state to another, for each of the two characters under eight different combinations of E(T) and SD(T), are summarized in Table 1. For both characters the average number of total transformations as well as state-to-state changes is higher with the faster rate prior, i.e. higher E(T). Thus, for carpel fusion the average total number of transformations changed from 1.39 (prior set at a low rate: E(T) = 1, SD(T) = 5) to 8.99 (prior set at a high rate: E(T) = 15, SD(T) = 5). If SD(T) is narrowed to one, the differences are even greater (1.27 to 14.01). Finally, averages for similar values of E(T) showed marked differences according to the different values of SD(T), except for two cases indicated in Table 1 (highlighted in), when the averages did not differ greatly.

**Table 1:** Average number of transformations estimated for each combination of the mean rate value (E(T)) and the level of confidence (SD(T)) estimated after 1000 simulations on the 201 last trees sample from the MCMC run.

	Rate parameter θ	Bias parameter Л	Total average transformations (95% HPD)	State-to-state transformations	
				0⇒1	1⇒0
*Pollen unit*					
Parsimony	—	—	8	6	2
E(T) = 1 SD(T) = 1	3.12	0.49	10.21 [8–15]	7.06	3.34
E(T) = 1 SD(T) = 5	8.16	0.49	22.06 [8–40]	12.26	9.81
E(T) = 5 SD(T) = 1	5.33	0.49	13.59 [8–21]	8.36	5.23
E(T) = 5 SD(T) = 5	7.79	0.49	20.82 [8–42]	11.66	9.15
**E(T) = 10 SD(T) = 1**	**9.96**	**0.49**	**26.6 [16–38]**	**14.24**	**12.31**
**E(T) = 10 SD(T) = 5**	**9.56**	**0.49**	**24.04 [8–44]**	**13**	**11**
E(T) = 15 SD(T) = 1	14.84	0.49	39.43 [26–52]	20.16	19.23
E(T) = 15 SD(T) = 5	12.6	0.49	32.66 [11–55]	17.04	15.62
*Carpel fusion*					
Parsimony	—	—	1	1	0
**E(T) = 1 SD(T) = 1**	**0.97**	**0.49**	**1.27 [1–3]**	**1.15**	**0.12**
**E(T) = 1 SD(T) = 5**	**1.05**	**0.49**	**1.39 [1–3]**	**1.2**	**0.19**
E(T) = 5 SD(T) = 1	4.65	0.48	3.48 [1–8]	2.18	1.31
E(T) = 5 SD(T) = 5	2.09	0.49	1.91 [1–5]	1.45	0.46
E(T) = 10 SD(T) = 1	9.72	0.44	8.36 [1–16]	4.39	3.97
E(T) = 10 SD(T) = 5	5.42	0.47	4.22 [1–11]	2.57	1.78
E(T) = 15 SD(T) = 1	14.77	0.4	14.01 [5–25]	6.9	7.11
E(T) = 15 SD(T) = 5	10.3	0.44	8.99 [1–20]	4.65	4.34

The maximum parsimony numbers of transformations were taken from a single most parsimonious tree arbitrarily chosen out of the seven found. The bold values represent a centred posterior distribution around the mean rate as visualized with the posterior distribution graphs.

**Figure 2:**
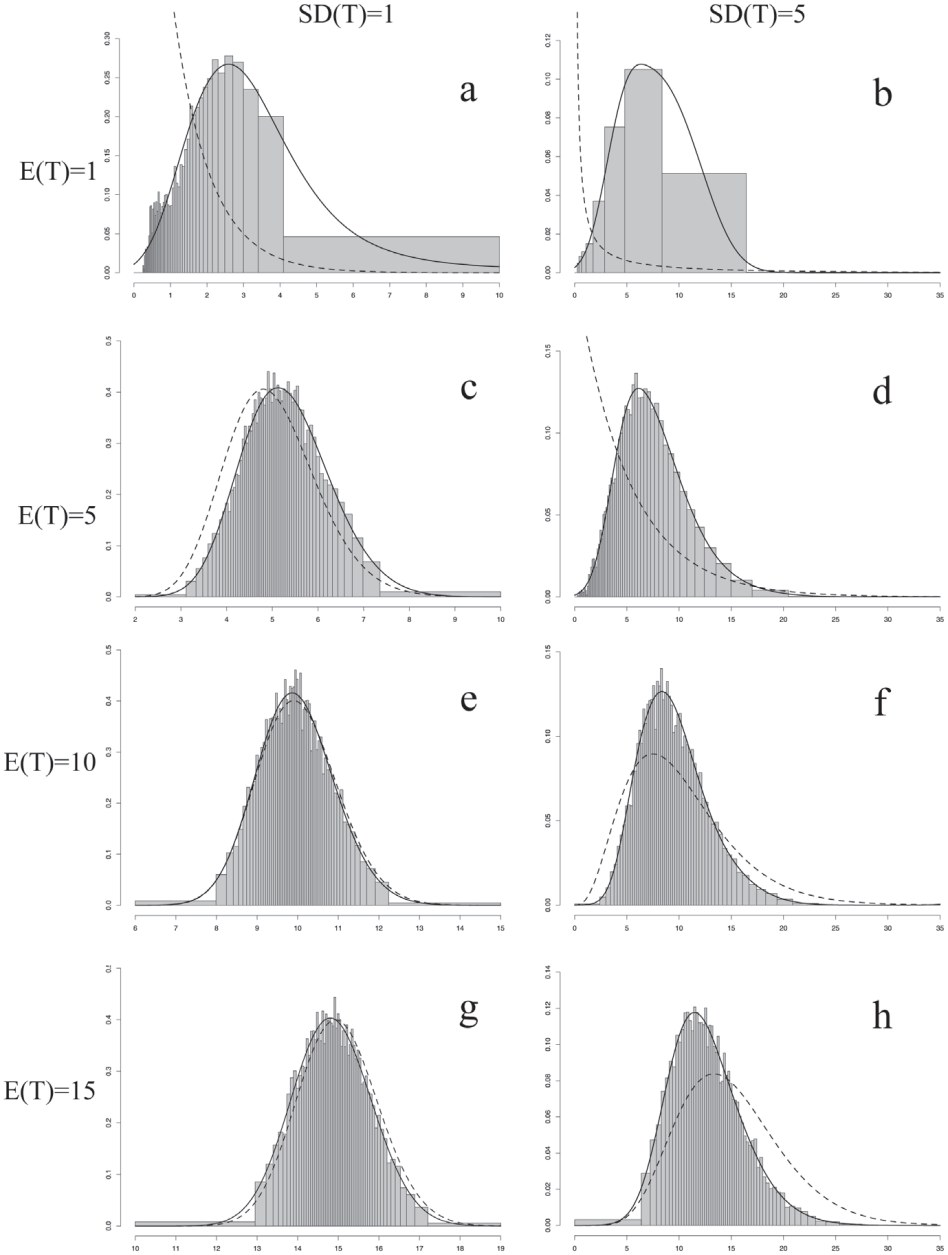
Posterior probability density distributions of the transformation rate θ for the pollen unit character. Posterior probability density distributions of each rate category given each combination of E(T) and SD(T) for the pollen unit character. The bars of the histogram represent the posterior distribution densities given the prior and the data for each rate category. The continuous gamma distribution was made discrete by breaking it into 60 equally probable rate categories [33]. Each category is represented by the mean of the portion of the gamma distribution included in the rate category. The total area of the histogram as well as the prior distribution equal one. The posterior density histograms are overlaid with the corresponding prior gamma density distribution (dashed) as well as a fitted curve (black). x-axis: rate of transformation. y-axis: density scale.

**Figure 3:**
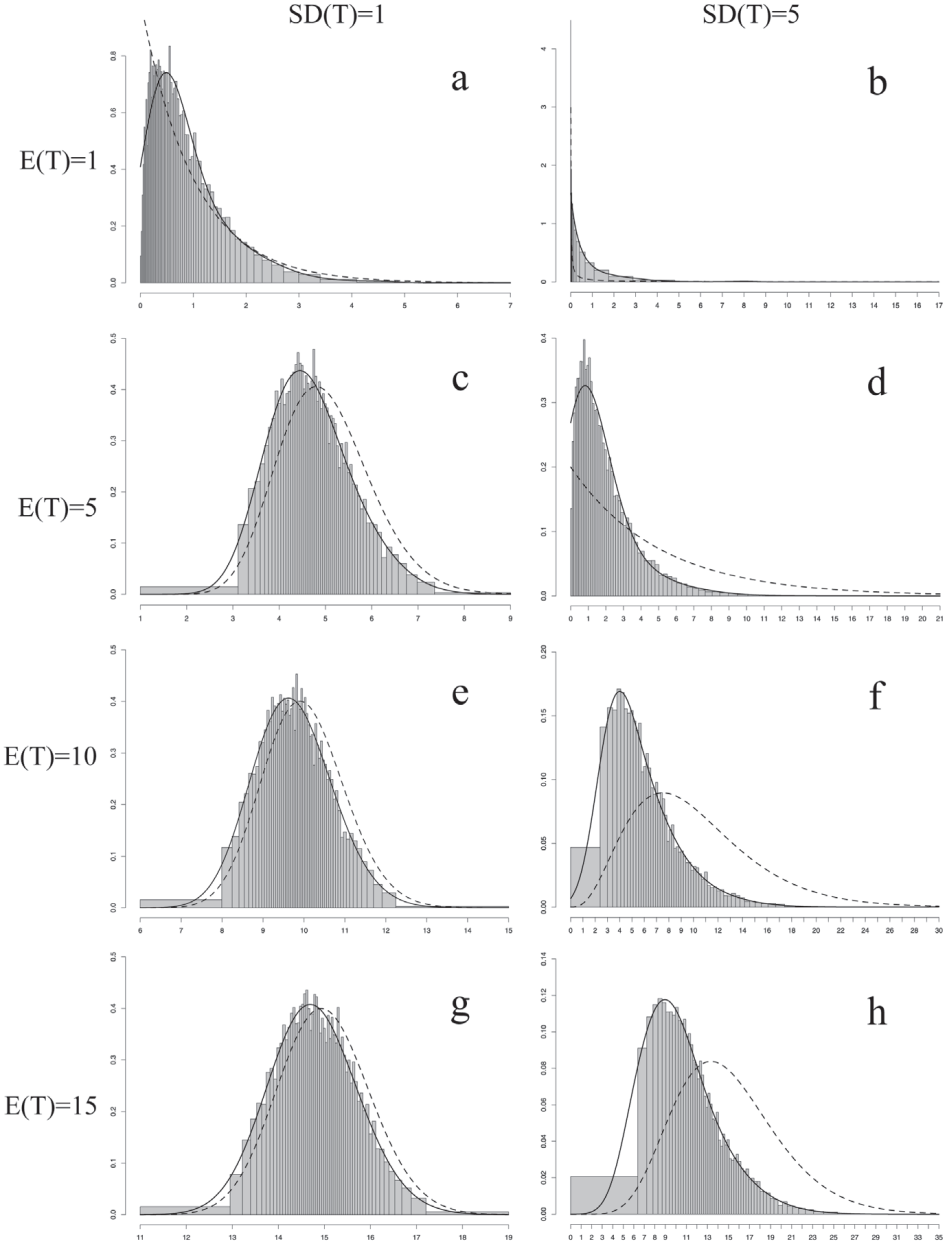
Posterior probability density distributions of the transformation rate θ for the carpel fusion character. Posterior probability density distributions of each rate category given each combination of E(T) and SD(T) for the carpel fusion character. The bars of the histogram represent the posterior distribution densities given the prior and the data for each rate category. The continuous gamma distribution was made discrete by breaking it into 60 equal probable rate categories [33]. Each category is represented by the mean of the portion of the gamma distribution included in the rate category. The total area of the histogram as well as the prior distribution equal one. The posterior density histograms are overlaid with the corresponding prior gamma density distribution (dashed) as well as a fitted curve (black). x-axis: rate of transformation. y-axis: density scale.

**Figure 4:**
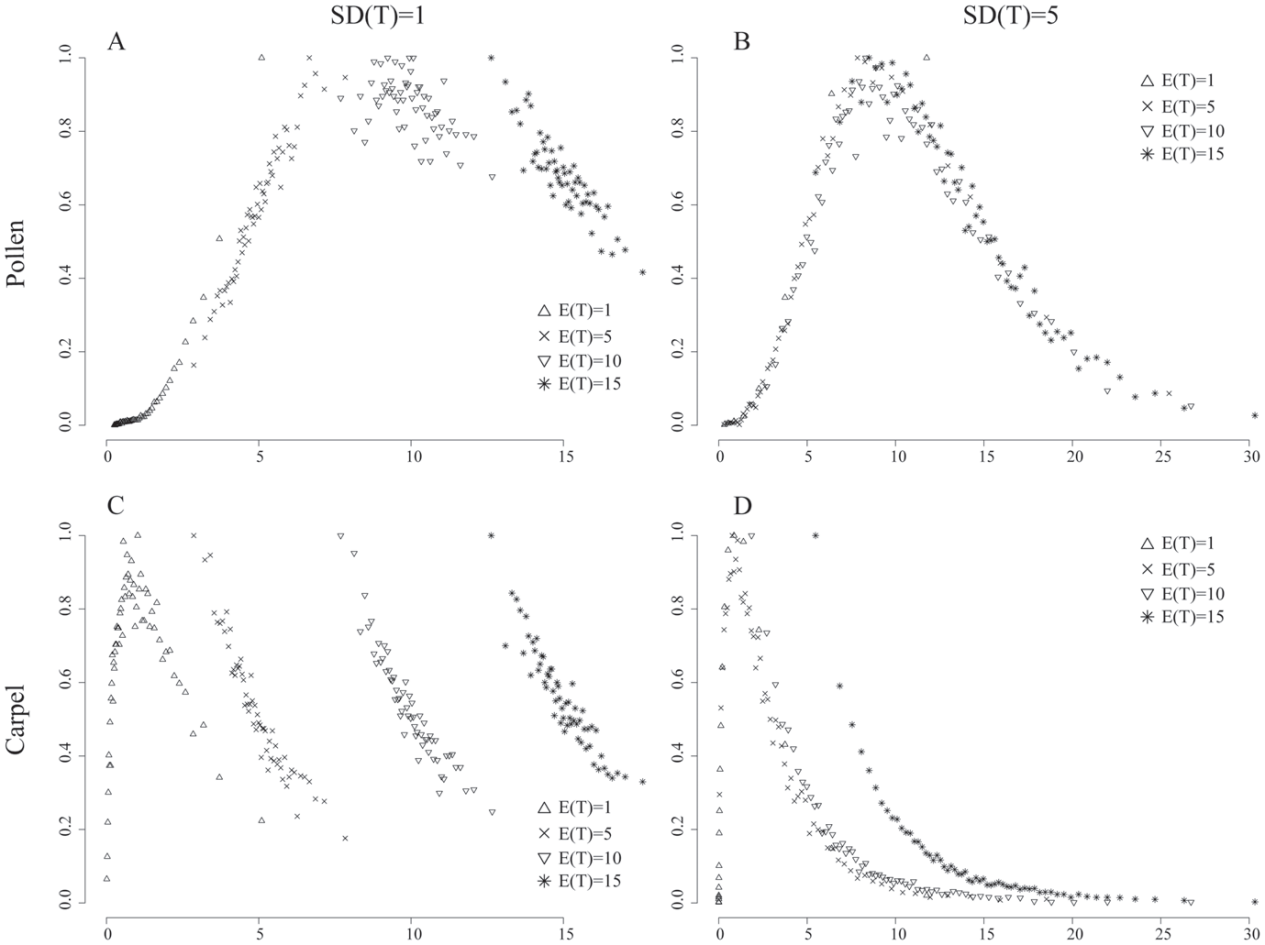
Posterior probabilities for both pollen unit and carpel fusion characters. Posterior probabilities for each rate prior E(T) given a standard deviation S(T) for both pollen unit and carpel fusion characters. x-axis: rate of transformation. y-axis: sampling frequency ( = posterior probability) of each discrete rate category.

The posterior density distributions of the transformation rate θ are presented in Figures 2 (pollen unit character) and 3 (carpel fusion character), while the posterior probabilities of the different rate categories are illustrated in Figure 4. For all graphs, the x-axis represents the rate value. For Figures 2 and 3, the x-axis is broken into 60 rate categories and their respective widths are illustrated. However, for a few combinations of E(T) and SD(T) the mean value of the range of some categories was extremely small (<1×10^−5^). As a result, these categories were never sampled during the simulation. Those categories were assigned a rate and sampling value of zero, resulting in less than 60 categories being represented. For Figure 4, each point represents the frequency at which the mean value of the each width was sampled after 10,000 simulations, interpreted as the posterior probability of each rate category to be sampled during a full analysis.

As expected, the mean rate and confidence values ((E(T) and SD(T), respectively) have an effect on the parameter space sampled, which is clearly visible when comparing the range of values between the different x-axes (Figs. 2 and 3). The mean rate value E(T) determines where in parameter space the values are sampled while the confidence SD(T) designates the extent of the range. When the confidence was high (SD(T) = 1; a, c, e, g in Figs. 2 or 3) the range of rate values sampled in parameter space was narrow, for example between 0 and 10 for E(T) = 1. In contrast, with a low confidence, the values that were sampled encompassed a wider range of rate values between 0 and 35 (SD(T) = 5, b, d, f, h in Figs. 2 and 3).

The posterior density histograms (Figs. 2 and 3) for each combination of E(T) and SD(T) are overlaid with the corresponding prior gamma density distribution (dashed) as well as a fitted curve (black). For both characters, the posterior densities are always influenced by the priors used. This is obvious when looking at the fitted curves, as they shift from left to right when going from E(T) = 1 to E(T) = 15 independent of the SD(T) specified.

Certain prior values, however, did provide a better match between the prior and fitted distributions. This was the case for the higher rate values for the pollen character (E(T) = 10 or 15; Fig. 2 e–h), or the lower rate values for the carpel character (E(T) = 1; Fig. 3 a, b). For both characters and for any other combination of priors tried, the posterior density histograms did not fit the prior gamma distributions very well (Fig. 2 a–d; Fig. 3 c–h).

The actual sampling frequency (i.e. posterior probability) of each rate category out of the 10,000 draws is shown in Figure 4 for both characters. In all cases under a low confidence SD(T) = 5 (except under E(T) = 15 for the carpel character), the categories around the same rate values were more thoroughly sampled than any other region, independent of the prior combination used (Fig. 4 b and d). For the pollen unit the highest sampling frequency was found for the categories around the rate value of 10 (Fig. 4 b). For the carpel fusion character this rate value was around 1 (Fig. 4 d). When a high confidence was specified (SD(T) = 1), the shape of the frequency distributions changed with different mean rates. For the pollen unit, the higher rate categories of the range were the most sampled under low and medium mean rates, giving a skewed shape to the distribution (Fig. 4 a). For E(T) = 15, the lower rates categories of the range were more sampled. For the fast mean rate (E(T) = 10), each category was more evenly sampled (Fig. 4 a). Inversely, for the carpel fusion character, the lower categories were always the most sampled under fast and medium mean rates (Fig. 4 c).

### Character History Space and Transformation Bias

**Figure 5:**
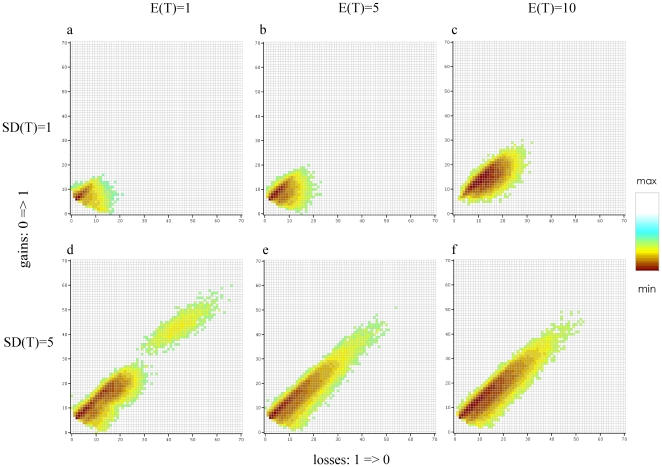
Posterior probabilities for all inferred character histories for the pollen character. Negative logarithm (base 10) of the posterior probabilities for all character histories that have occurred during the simulation for the pollen character and the combinations E(T) = 1, 5 and 10 and SD(T) = 1 and 5. The x-axis represents the total number of transformations from 1 to 0 (i.e. number of gains) and the y-axis from 0 to 1 (i.e. number of losses). It is important to note that as we used the negative logarithm thus the lowest values (dark red) represent the highest PP_c_s. The colours for the PP_c_ are not the same across the graphs, as they represent the values for each independent analysis.

**Figure 6:**
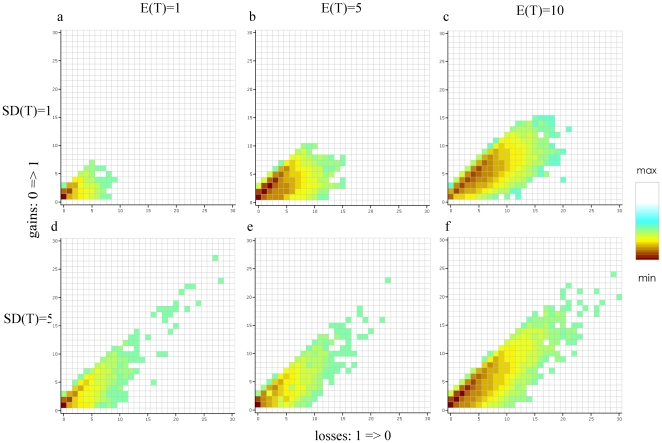
Posterior probabilities for all inferred character histories for the carpel fusion character. Negative logarithm (base 10) of the posterior probabilities for all character histories that have occurred during the simulation for the carpel fusion character and the combinations E(T) = 1, 5 and 10 and SD(T) = 1 and 5. The x-axis represents the total number of transformations from 1 to 0 (i.e. number of gains) and the y-axis from 0 to 1 (i.e. number of losses). It is important to note that as we used the negative logarithm thus the lowest values (dark red) represent the highest PP_c_s. The colours for the PP_c_ are not the same across the graphs, as they represent the values for each independent analysis.

The exploration of character history space by the 201,000 realizations is shown in Figures 5 and 6, split according to the different combinations of E(T) and SD(T). These figures simultaneously represent all the different character histories, their respective frequencies, and the transformation bias, as explored during the analysis. The character history space explored for the pollen unit (Fig. 5) is much larger than for carpel fusion (Fig. 6), visible from the difference in number of gain/loss combinations. In both cases the space explored by the simulation is larger under a low confidence (SD(T) = 5; Fig. 3 d–f and Fig. 6 d–f) than under a high confidence (SD(T) = 1; Fig. 5 a–c and Fig. 6 a–c). In other words, faster transformation scenarios are sampled when our confidence is low and this is independent of the mean rate prior used. However, a large majority of the character histories occur only a few times (low PP_c_), which is indicated by the bright yellow and green colours. The highest posterior probabilities (dark red squares) are returned for character transformation scenarios that are slightly biased towards gains (0⇒1), as these are positioned above the diagonal in all plots. In contrast, the character history space that is explored is skewed towards a slight excess of losses over gains. This pattern is similar for all plots in Figures 5 and 6.

The different prior values had a contrasting influence on the identification of the most probable character history. For carpel fusion, the same character history (1 gain and 0 losses; Tables 2 and 3, Fig. 6) was assigned the highest PP_c_ independent of the priors used (except in three extreme cases, Table 3, Fig. 6 c). This is graphically visible in Figure 6 where the darkest red square is mainly situated at 1 gain and 0 losses. However, for the pollen unit, different values of E(T) always return different most probable character histories (Tables 2 and 3, Fig. 5). Moreover, with SD(T) = 5, many alternative character histories received an almost equal PP_c_ value (Table 2). This is also visible in Figure 5 where numerous dark-red squares cover a large number of squares. Finally, in Figure 5 d the cloud is broken in two at around 30 transformations from 0 to 1 and 1 to 0. The squares above 30 represent very high rates of transformation (>60 transformations over the tree).

**Table 2:** The first six character histories with the highest posterior probability (PP_c_) for each combination of E(T) = 1 and 5 and their respective SD(T).

	Pollen unit			Carpel fusion		
	0⇒1	1⇒0	PP_c_	0⇒1	1⇒0	PP_c_
E(T) = 1 SD(T) = 1	6	2	0.307	1	0	0.867
	7	3	0.157	2	1	0.063
	7	2	0.103	2	0	0.042
	8	4	0.075	3	2	0.007
	8	3	0.041	1	1	0.007
	6	3	0.036	3	1	0.005
E(T) = 1 SD(T) = 5	6	2	0.062	1	0	0.848
	7	3	0.056	2	1	0.067
	8	4	0.049	2	0	0.036
	9	5	0.042	3	2	0.015
	10	6	0.032	1	1	0.006
	11	7	0.021	3	1	0.005
E(T) = 5 SD(T) = 1	7	3	0.118	1	0	0.295
	8	4	0.115	2	1	0.285
	9	5	0.084	3	2	0.145
	6	2	0.066	4	3	0.055
	10	6	0.05	2	0	0.035
	8	5	0.044	3	1	0.033
E(T) = 5 SD(T) = 5	7	3	0.06	1	0	0.695
	6	2	0.056	2	1	0.138
	8	4	0.052	2	0	0.045
	9	5	0.042	3	2	0.04
	10	6	0.033	3	1	0.013
	11	7	0.027	4	3	0.013

**Table 3:** The first six character histories with the highest posterior probability (PP_c_) for each combination of E(T) = 10 and 15 and their respective SD(T).

	Pollen unit			Carpel fusion		
	0⇒1	1⇒0	PP_c_	0⇒1	1⇒0	PP_c_
E(T) = 10 SD(T) = 1	14	10	0.037	4	3	0.134
	13	9	0.035	3	2	0.13
	15	11	0.033	5	4	0.105
	12	8	0.03	2	1	0.088
	14	11	0.029	6	5	0.066
	13	10	0.027	7	6	0.034
E(T) = 10 SD(T) = 5	9	5	0.03	1	0	0.3
	10	6	0.029	2	1	0.22
	8	4	0.029	3	2	0.125
	11	7	0.027	4	3	0.065
	7	3	0.025	5	4	0.032
	12	8	0.024	2	0	0.031
E(T) = 15 SD(T) = 1	39	63	0.020	6	5	0.074
	39	6	0.019	5	4	0.069
	37	75	0.019	7	6	0.064
	37	57	0.019	4	3	0.051
	36	52	0.018	8	7	0.050
	36	45	0.018	9	8	0.034
E(T) = 15 SD(T) = 5						
	14	10	0.016	3	2	0.120
	13	9	0.016	2	1	0.112
	12	8	0.015	4	3	0.103
	15	11	0.015	5	4	0.074
	16	12	0.014	1	0	0.062
	11	7	0.014	6	5	0.050

## Discussion

### Influence of the prior gamma distribution on θ

It has been shown that priors do influence Bayesian inference in phylogenetic reconstruction [31], [32]. Within the realm of Bayesian character evolution little work has been done on the influence of priors on the results. Pagel et al. [2], under a slightly different model of character evolution (see above), showed that alternative priors could lead to different results, but the reasons for this were not discussed in details. Within the method of posterior mapping, the role played by the prior gamma distribution on the transformation rate θ in posterior mapping is still largely unclear [4], [9]. Our study showed that the prior distribution on the rate parameter θ had a significant impact on the results for both characters analysed. The differences were obvious in the two results reported here: the average number of transformations of character states (Table 1) and the most probable character history (Tables 2 and 3). In the latter case, the prior had a stronger impact on the pollen character than on the carpel fusion character (Tables 2 and 3) and will be discussed later. In the former, the different combinations of E(T) always produced different outcomes (Table 1). These differences are important to stress as they would result in alternative evolutionary interpretations of the morphological character(s) under study. Under a high confidence (SD(T) = 1), the values of the continuous-time Markov chain are drawn from significantly different regions in parameter space and the posterior distributions are generally skewed (Fig. 4, a and c). If different regions are sampled with a changing E(T), different character histories will be realized thus leading to the different observed averages. Under a low confidence regime (SD(T) = 5) the explanation of the observed averages is more intriguing because the parameter space sampled between alternative prior values is equivalent (Fig. 4 b and d). Additionally, the same region of parameter space is systematically more sampled than others and this is independent of the prior used (around 10 for the pollen unit and 1 for the carpel fusion). However, the resulting number of transformations is clearly different for the alternative rate priors used (Table 1). These differences can be explained from two observations. First, this “same” parameter space is not equally sampled. For example in figure 4 b, for E(T) = 1 sampling is denser around the lower rate values (left), while for E(T) = 15 sampling is denser around the higher rate values (right). Only for E(T) = 10 is the sampling evenly spread out in a normal fashion across parameter space. The second less obvious observation for explaining the different results is the width of the rate categories that are generated with the different priors, or discretization. Discretization concerns the process of transferring a continuous distribution (or models) into discrete counterparts. In our specific case, the continuous gamma distribution is discretized (i.e. broken) into 60 equally probable categories, i.e. each category has an equal surface area [4], [33]. This effectively means: small widths around the mean and increasingly larger widths away from the mean (the marginal regions). Each category is then assigned a fixed rate value equal to the mean of the range. Thus, these widths are directly dependent on the shape of the prior probability distribution, and therefore on the values assigned to the parameters α_S_ and β_S_. It is the generation of different width ranges within an equal parameter space for different prior values that also produce the observed disparities in the average number character transformations. For example, for the pollen character, categories around the rate value 10, which is the most frequently sampled category for any value of E(T), present large widths for E(T) = 1 (Fig. 2 b), medium widths for E(T) = 5 (Fig. 2 d) and smaller widths for E(T) = 10 (Fig. 2 f). Because each width is represented by its mean, the different discretizations around the most frequently sampled rate value will have a direct effect on the average number of transformations as shown by the results.

Finally, discretization is also responsible for another counterintuitive result. For the pollen unit, a higher average number of transformations was returned under the small mean rate prior when compared to the medium one (Table 1), although we would expect a lower average for the small rate. Figure 2 b shows the ranges of the two last rate categories generated under E(T) = 1 and SD(T) = 5, which encompassed a large range of values and were represented by their mean of 11.78 and 32.24. The latter rate value is the largest rate category generated. Although it had a low posterior probability (sampled 65 times out of 10,000 draws), this was still enough during the course of a long simulation to produce a few high transformations (>60). These high transformations are clearly visible in Figure 5 d, where character history space is split into two at around 30 gains and 30 losses. These high generated transformations are responsible for returning an average superior to the one for E(T) = 5. For carpel fusion, this broken cloud effect is also visible but to a lesser extent (Fig. 6, broken at 15 gains and 15 losses), and was not marked enough to produce a superior average than for E(T) = 5 (Table 1). The broken cloud effect seems to be related to mean rate values close to zero coupled with high standard deviations and requires further investigation.

The main conclusion is that priors will exert an indirect and noticeable effect on the results. These results are significant as the average number of transformations are the main results provided by SIMMAP and are the ones that are generally reported and used for the interpretation of character evolution, e.g. [13].

### Levels of Homoplasy

Although we have shown that the prior distribution on the transformation rate will affect the average number of transformations, we have also shown that it had a contrasting influence when identifying the most probable character history (Tables 2 and 3). For carpel fusion, the same character history was always assigned the highest PP_c_ independent of the prior used, except with extremely unrealistic prior combinations (Table 3). On the other hand, for the pollen unit, different character histories were most probable between the different values of E(T) as well as within the same analysis (several sub-equally probable character histories, Tables 2 and 3). These differences are also visible in the character history space when using the appropriate prior values. For the pollen character the red squares (indicating a high PP_c_) cover a much wider space (Fig. 5 c and f) than for the carpel fusion character which is concentrated around one gain - zero losses (Fig. 6 c and f). One explanation for these differences is the level of homoplasy present in the data for each character. A character's consistency index *c_i_* [34] provides a simple measure of the overall homoplasy of the character [35]. For pollen unit and carpel fusion the *c_i_* was 0.13 and 1.0, respectively. Thus, the character with high levels of homoplasy (a low *c_i_*) had several equally most probable histories, and when the *c_i_* of the character was high, a single most probable history was significantly favoured. Homoplasy is positively correlated with the rate of evolution of a character [36], [37]: the higher the rate, the lower the *c_i_*. For fast evolving characters, the levels of homoplasy will always be high and thus many equally most probable character histories will be found. This appears to be the case for the pollen character used here. However, this relationship might not always be straightforward. For example, the characters studied in Huelsenbeck et al. [4], seastars with or without larval feeding [38] and, absence or presence of a horned soldier in aphid species [39], were also shown to have a high rate of transformation amongst states (E(T) = 10), as judged from their posterior distributions. In addition, the characters had a relatively low *c_i_* (0.33 for the aphid dataset, and 0.25 for the seastar dataset). However, even under two contrasting mean rate priors (E(T) = 1 and 10), the same character history had the highest PP_c_: four gains and zero losses for larval feeding, one gain and two losses for horned soldiers. In this case, despite relatively high levels of homoplasy, one character history was significantly favoured over the others. Thus, the relationship between homoplasy and rates of evolution is complex as noted by Sanderson and Donoghue [35]. In the examples provided by Huelsenbeck et al. [4], it would appear that even though the characters were fast evolving and likely to be more homoplasious, the signal provided by the data was strong. In that case the priors seemed to have little influence on identifying the most probable character history as has been previously suggested for Bayesian analyses in general, e.g. [11]. It is important to stress that this concerns the most probable character history, a result not immediately available when using SIMMAP. The exact influence of the level of homoplasy and the strength of the data on the outcome of the analysis is still unclear. Further simulation studies should be undertaken in order to address this question.

### Specification of the gamma prior distribution on θ

Choosing appropriate priors for Bayesian analyses is a hard task, inciting ongoing debate [40], [41], [42]. How should one specify the parameters of the prior gamma distribution in posterior mapping? Although our results are based on just one case study, we think that they do provide useful insights into how priors can influence the outcome of a posterior mapping analysis. First, there is a prior mean rate (E(T)) that better suits the data than others. For example, in all cases with SD(T) = 5, the sampling frequency was maximal roughly around the same rate value (Fig. 4 b and d), regardless of the E(T) (just under 10 for the pollen unit and around 1 for carpel fusion). These values could be interpreted as the “appropriate” mean rate for the character given the data. These appropriate values are also found when the confidence level is changed. Under a high confidence (e.g. a low SD(T)) we narrow down the possible parameter space. In that case a skewed posterior distribution could result in only part (or none) of the “true” parameter space being sampled. Second, we also observed that when the appropriate transformation rate was selected, little difference was observed between a high and a low confidence (SD(T) = 1 or 5) on the average number of transformations (Table 1). This implies that the mean of the prior rate value (E(T)) is more important than the associated standard deviation (SD(T)). Thus, in agreement with Huelsenbeck et al. [4], it would seem that the data contains some information over the rate of transformation and that a prior distribution generating a skewed posterior probability distribution is not appropriate given the data. Finally, as we have shown in Figure 4 b and d and Table 1 choosing a large standard deviation in order to cover all parameter space coupled with an arbitrary rate is not the appropriate solution, because uneven sampling and discretization of this parameter space will still influence the results.

The idealized Bayesian approach dictates that one should choose priors by using external knowledge independent of the data at hand. In some cases choosing a prior value on θ could be fairly straightforward. This would be the case for a character such as carpel fusion. Syncarpy was inferred to have evolved once with no losses or c. four times with four losses within Annonaceae (Table 1). Our prior knowledge suggests however that an evolutionary scenario of four gains is highly improbable and unrealistic because syncarpy has rarely evolved in magnoliids with reversals being even rarer [25], [27]. In contrast, for some characters prior knowledge would not be able to clearly indicate which result to expect. We can be confident that the pollen character evolves faster than the carpel character (an independent dataset suggested high homoplasy [29]), but we would be unable to favor one prior value over the other in a well-informed way (Table 1). In such cases, specifying prior values can be problematic. One alternative is to adopt an empirical Bayes estimator approach whereby the data is used to estimate the parameters of the gamma prior distribution on θ. The empirical Bayes estimator approach has been used under different models of Bayesian character evolution. For example, to define the beta prior distribution placed on the rate parameter, Pagel et al. [2] used three different prior values, two of which were inferred from the data at hand. Indeed the parameters were estimated either via maximum likelihood values or via the likelihood surface [2]. Such an approach would seem to reduce the problem of prior choice, but introduces problems of its own [43] that we shall not address here. Whatever the method used to define the prior values, we suggest checking the resulting posterior distributions in order to make sure they are evenly sampled around the specified mean (normal distribution) and not centred on the highest or lowest categories (skewed distribution). Although we acknowledge that a normal posterior distribution is not always the best way to represent uncertainty, we do think that because of the discretization method used (see above), a skewed distribution will lead to erroneous reconstructions. The choice of the normal distribution is mainly based on the way parameter space is discretized, which is centred on the mean. Finally, it will be important to report the values of the priors used during any study as this will allow others to repeat the analysis under different prior assumptions.

Many more questions remain to be answered and especially *how* exactly will the priors affect the results in certain situations still needs to be explored: for example how will the priors affect the results with different degrees of homoplasy, rate heterogeneity of a morphological character, tree shape, sampling of taxa and characters. Answering these questions using simulated data would allow for a better understanding of the precise role priors play in posterior mapping.

## Materials and Methods

### Phylogenetic analyses

The results presented here are derived from a DNA sequence data matrix of the Annonaceae family [28] totalling 66 taxa sampled across the family. The dataset was composed of six chloroplastic markers, three non-coding (*trnL-trnF*, *trnS-trnG* and *psbA*-*trnH*) and three coding (*ndhF*, *rbcL* and partial *matK*), totalling 7945 characters. Gaps were coded as separate characters. All phylogenetic analyses were run using the Metropolis-coupled Monte Carlo Markov chain (MCMCMC) algorithm implemented in MrBayes, ver. 3.1.2 [44], under the best partitioning strategy identified using the Bayes factor [45] and following Brandley et al. [46]. For each partition, the best performing evolutionary model was identified using the Akaike information criterion (AIC [47]) using MrModeltest [48]. Three separate runs (with one cold and three hot chains) of five million generations each were undertaken and stationarity as well as convergence between the MCMC runs was checked using both Tracer v. 1.3 [49] and the online program AWTY [50].

### Influence of the Transformation Rate Prior

The impact of alternative prior distributions on the transformation rate (θ) was studied by subjecting the carpel fusion and pollen characters to the posterior mapping method as implemented in the program SIMMAP version 1.0 beta 2.3 (build 12092006 [19]). Both characters were scored for each taxon following Couvreur et al. [28]. Both characters were unordered.

SIMMAP allows the user to specify two parameters (α_S_ and β_S_) that define the prior gamma distribution placed on the transformation rate θ and one parameter (α_B_) for the beta distribution placed on the directional bias Л. For the latter, a flat prior was used in all analyses (α_B_ = β_B_ = 1). To compare the effect of the prior distributions on θ we must be sure to compare them equally, i.e. make sure they have either the same mean (E(T)) or standard deviation (SD(T)). For the prior gamma distribution we have 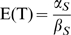 and 
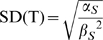
 [4], [33]. Formulating these equations as a function of α_S_ and β_S_ leads to 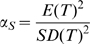 and 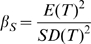. This formula allows us to find the values of the parameters α_S_ and β_S_ for any required combination of E(T) and SD(T).

The simulation of the continuous-time Markov chain was realized 1,000 times over the last 201 trees imported from the MrBayes analysis for both characters. Eight different combinations of E(T) and SD(T) for the prior distribution on θ were analysed. A slow, medium, fast and very fast mean rate (E(T) =  1, 5, 10 and 15 respectively) were each combined with a high and low confidence (SD(T) = 1 and 5, respectively). Note that these values and associated terminology were chosen relative to the biology of the morphological characters considered for this specific analysis. The total number of character transformations, and the number of transformations between each state were averaged over all realizations. Finally, the actual number of times a particular character history occurred throughout the 201,000 realizations was calculated (for example, how many times was “one gain and one loss” simulated). A perl script (Vriesendorp and Couvreur, unpublished) was written in order to extract that information from the SIMMAP output files. Dividing the number of occurrences by the total number of realizations gives the posterior probability of each character history (abbreviated as PP_c_, not to be confused with the PP of the nodes in the phylogenetic tree). To reduce the large range of values between PP_c_s (5^e−6^ to 0.9) the negative logarithm of the PP_c_ for each of the characters histories was plotted on a 2D graph using Kyplot (Koishi Yoshioka, v.2 beta 15, www.woundedmoon.org/win32/kyplot.html).

We also evaluated the influence of the priors on the posterior distribution. The posterior distribution for each combination of E(T) and SD(T) was estimated by undertaking 10,000 realizations using the “number of realizations sampled from priors” function in SIMMAP. This approach estimates the posterior distribution of the parameters by sampling the prior without undertaking the full length analysis. The posterior distribution for each combination was then visualized in Tracer v. 1.3 [49] by converting the SIMMAP output file to a Tracer file using the python script “convert2tracer.py” found on the SIMMAP website (Bollback, http://www.simmap.com). The prior gamma distribution was broken or discretized into 60 rate categories, each of which represents an equal probability density [33]. As the areas under the probability curve are equalized, the resulting categories have different widths. For each combination of E(T) and SD(T) two different graphs were produced. A posterior density histogram was normalized by dividing the number of counts within each rate category by the width of that category, with the total surface area of the rectangles equalling one. This representation allows for the overlay of the prior gamma probability density as a reference. Smooth curves were fitted using the software in R and based on Eilers [51]. Moreover, this provides a visualization of how parameter space is discretized (broken down) given each combination of E(T) and SD(T).

A second graph was generated that represents the number of times each rate category (represented by its mean) was sampled out of 10,000 draws, i.e. the posterior probability of each category following Huelsenbeck et al. [4]. This graph differs from the previous one in that we no longer take the width of the rate into account, and by overlapping the posterior distribution for each E(T) we can clearly identify the regions of parameter space that are more sampled than other under the alternative priors chosen.

Maximum parsimony optimization results were also provided as a reference only. The majority rule consensus tree from the Bayesian analysis was used for subsequent analyses using Mesquite v. 1.11 [52]. Both characters were treated as unordered.
